# A systematic review of machine learning algorithms for mortality risk, readmission and phenotype prediction in patients with heart failure: exploring key data sources, input variables and outcomes

**DOI:** 10.1186/s12911-026-03560-8

**Published:** 2026-06-03

**Authors:** Aleksandra Flok, Patricia Kajüter Rodrigues, Sofiya Pohurskyy, Frank Teuteberg

**Affiliations:** Accounting and Information Systems, Osnabrück University, Katharinenstr. 1, 49074 Osnabrück, Germany

**Keywords:** Heart Failure, Machine Learning, Prediction, Mortality, Readmission, Phenotypes

## Abstract

**Background:**

Heart failure is not only a prevalent disease with a high mortality rate, but also generates high costs for healthcare systems. By training artificial intelligence (AI) models on medical data, it is possible to predict changes in health status that may lead to hospital readmissions or death. Such predictions enable better patient care and a proactive response to deterioration.

**Methods:**

We conducted a systematic literature review of relevant AI studies using multiple machine learning (ML) algorithms to predict readmission and mortality in heart failure in previously diagnosed patients as well as clustering phenotypes. We synthesized and categorized the studies by the outcome variables, i.e. mortality, readmission, and phenotyping. The Scopus database was searched in September 2024 for relevant studies published between 2014 and 2024. Studies were included if they focused on heart failure, used data from electronic health records or hospital records, adopted machine learning techniques, analyzed readmission, mortality or phenotypes, and included patients no younger than 18 years of age.

**Results:**

A total of 109 relevant studies were identified. In the mortality group (68 studies), age, serum creatinine level, serum sodium level, systolic blood pressure and blood urea nitrogen were among the most frequently mentioned relevant variables for predicting mortality. Comorbidities, blood urea nitrogen and age were identified as the most relevant variables for readmission (32 studies). The remaining studies dealt with phenotyping or further outcomes. Within all groups, random forest was the most recommended ML algorithm for prediction, followed by support vector machines. Nine key implications were derived from this review to guide future research and practice in AI-based studies. These implications emphasize improving model generalizability, data quality, and explainability to enhance the robustness and effectiveness of AI applications.

**Limitations:**

We did not use formal tools to assess the risk of bias due to missing results but addressed potential reporting bias by documenting missing performance metrics as “not available” and prioritizing studies with comprehensive reporting to ensure transparency in data synthesis and interpretation.

**Conclusions:**

The review shows that it is useful to group the studies into outcomes of readmission, mortality and phenotyping. This made it possible to highlight the relevant variables in each group. In addition, the different predictive capabilities of each outcome were identified. This research was conducted within the scope of the project KardioInterakt, which is supported by the German Federal Ministry of Research, Technology and Space (BMFTR) (grant number 16SV8906).

**Clinical trial number:**

Not applicable.

**Supplementary Information:**

The online version contains supplementary material available at 10.1186/s12911-026-03560-8.

## Background

In 2020, the European Heart Network estimated that 5,000 people in the European Union (EU) are dying from cardiovascular disease every day. This translates into 1.8 million deaths per year from cardiovascular disease (CD). CDs affect an estimated 60 million people in Europe, with an additional 13 million new cases each year and is the leading cause of mortality at 36% [1]. Heart failure (HF) is a CD and is the focus of this paper. HF comes often with symptoms such as breathlessness, fatigue, and ankle swelling and is a functional abnormality of the heart that results in inadequate cardiac output. HF can be divided into NYHA (New York Heart Association) classes I to IV, which form a functional classification of symptoms and activities. The higher the NYHA class, the greater the restrictions on physical activity and the more severe the symptoms of HF [2]. In 2023, HF was the third leading cause of death among women and the seventh leading cause of death among men in Germany [3]. The aim of this paper is to investigate how machine learning approaches in heart failure research can improve prediction of mortality, readmission, and phenotyping outcomes.

The use of Artificial Intelligence (AI) in the field of HF involves a variety of data sources, such as electronic patient records (EPR), electrocardiogram (ECG) data, remote monitoring or data from wearables. AI can be used to provide a diagnosis of HF, a classification and phenotype, or a prediction or prognosis of health status [4–6]. Recommendations for therapy and personalized medicine are also a possible area of application [6]. Depending on the role of the algorithms, they have the potential to be used as clinical decision support tools or to identify and monitor patients, which is currently time-consuming for clinicians [7]. Using AI to predict mortality or hospital readmission in HF can help individualize patient care and manage the disease [8]. One example is early detection of HF patients at high risk for hospital readmission, which can improve HF outcomes and reduce healthcare costs [8]. By identifying phenotypes within a patient data set, differences in outcomes can be identified and used to guide future research or to select suitable treatment therapies [7]. A differentiating factor in the literature is what the AI models are intended to be used for. Some are aimed at diagnosing HF patients and determining whether HF is expected [9]. Others start their analysis at a later stage, when patients diagnosed with HF are already part of the sample and aim to predict their mortality risks or hospital readmissions [10, 11].

Jasinska-Piadlo et al. [12] reviewed relevant machine learning (ML) studies in the field of HF and were able to identify four clusters of outcomes: HF detection from electronic health record (EHR), mortality prediction, readmission, and classification. The majority of studies addressed the detection of HF. Mpanya et al. [13] completed a review of 30 studies that focused on predicting all-cause or cardiac mortality or all-cause or HF-related hospitalization in patients with HF. They extracted information from the studies on the study population, number of variables, data sources, use and validation of the AI model, and performance measures [13]. In their review, Azmi et al. [14] examined 41 studies on classification and prediction algorithms. The results of the review were structured according to the following content: proposed work, dataset, comparative and proposed algorithm, accuracy mentioned as a performance metric and limitations [14].

Identifying key factors influencing mortality, readmission, and phenotyping in heart failure is essential for improving machine learning-based prediction models and supporting clinical decision-making.To the best of our knowledge, ours is the first paper to explicitly address all these three outcomes and analyze them in the context of ML techniques. This leads to the following research questions (RQs):

### RQ1:

*Which variables are highly influential in predicting outcomes in phenotyping, readmission, and mortality risk in HF?*

### RQ2:

*What are the lessons learned from the application of ML techniques and 10 years of research in this field?*

The paper is structured as follows: The background section is followed by an outline of the methodological approach. The methodology section presents the systematic literature review and explains the selection of relevant studies. The results section describes and highlights the findings of the literature review. The discussion draws out implications for research and practice, and addresses limitations. Finally, the most relevant findings are summarized in a conclusion with regard to the RQs.

## Methods

**Fig. 1:**
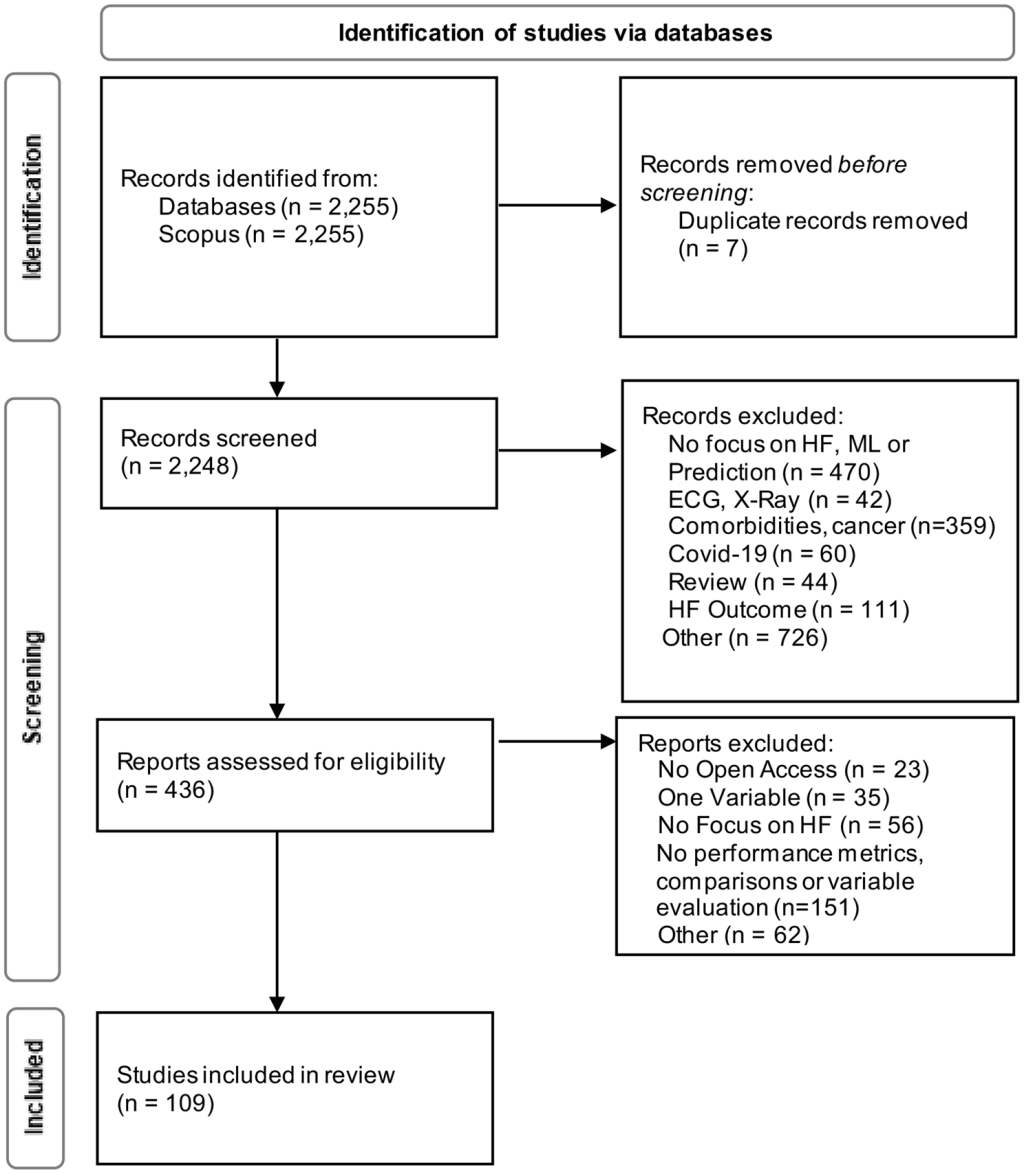
PRISMA Flow Chart

Our literature review aims to provide an overview of the current use of AI for the prediction of the mortality risk, readmission and phenotyping of HF. The systematic review follows the guidelines of Webster and Watson [15] and Niehaves et al. [16]. Additionally, we apply the methodological guidelines of PRISMA statement for the documentation [17]. This study is registered on PROSPERO with the registration number CRD420251035189. Cooper [18]’s taxonomy is used to frame the review. The focus of this literature review is to examine the research findings. The main aim is to summarize and integrate the findings of the relevant studies. The structure is a combination of a conceptual and methodological approach based on the research findings and applied ML techniques. To ensure objectivity, all authors worked on the literature review simultaneously. To decide whether to include a study or not, the authors conducted a workshop to discuss all relevant papers. A paper was included in the review if at least three out of four authors agreed on its relevance, reflecting a high level of consensus. Figure 1 documents the search process in a flowchart diagram. The Scopus database was used for the following search string: “heart failure” AND “predict*” OR “prevent*” OR “diagnosis” OR “risk*” OR “detection” AND “machine learning” OR “artificial intelligence”), which was applied to the title, abstract and keywords. The publications are published in English and between 2014 and 2024 to ensure actuality of the results. In 2015, only one study on the use of ML techniques for patients with HF was initially conducted, but interest in this research direction increased significantly from 2020 onwards, peaking in 2023 with 28 studies. In total, there were 2255 hits. After removing duplicates there were 2248 hits for the screening process. Next, the titles and abstracts were screened using the inclusion criteria, such as a focus on HF, data from EHRs, hospital records or similar, use of ML, and outcome variable is either phenotypes, readmission or mortality/survival risk. Further, only studies that examine data from patients over 18 years of age with a diagnosed HF condition and are available under open access were included. Exclusion criteria include studies that only apply statistical methods without using AI models, studies dealing with other diseases are also excluded. Studies using only ECG or X-Ray data as well as literature reviews are also among the exclusion criteria. Studies categorised under ‘HF Outcome’ were excluded because they only examined the presence or absence of HF, rather than applying predictive modelling over time for specific outcomes such as mortality or readmission. Articles grouped under ‘Other’ were excluded for various reasons, such as focusing on biomarkers (e.g. blood or saliva), subpopulations (e.g. pregnant women, children or animals) or using ML techniques solely for diagnostic rather than prognostic purposes.

After screening the title and abstract, a total of 474 hits were identified for further analysis. After applying the inclusion and exclusion criteria to the full-text. A total of 109 studies were included in the literature analysis, which could be grouped into readmission, mortality, phenotyping and other outcomes. The Prisma flowchart using the template of Page et al. [19] documents the literature selection.

## Results

The individual outcomes were extracted from all relevant records. In some cases, authors are listed more than once in Table 1 if they have analyzed both hospital readmissions and mortality or if they have examined several time periods simultaneously. The extracted outcomes show the time period over which the applied ML algorithms are able to make a prediction. For example, mortality outcomes are examined over different time periods, starting with in-hospital deaths and extending to 2 days, 7 days, 30 days, 3 months, 6 months, 1 year, 3 years and 6 years. Similarly, readmission outcomes are monitored at various intervals, including 30 days, 100 days, 3 months, 6 months, 1 year, 3 years and 6 years. Of the 10 studies that performed phenotyping or clustering, 7 used unsupervised learning, 2 used supervised learning and 1 used semi-supervised learning.

**Table 1:** Overview of prediction classifications of relevant studies

	Classification	
Mortality Risk	In hospital/General Risk Prediction	[11, 20–43]; [44–63]
	≤30 days	[59, 64–70]
	<1 year	[45, 66, 68, 71–74]
	≥1 year	[34, 59, 66, 68, 70, 75–88]
Readmission outcomes	General risk prediction	[89, 90, 10, 76, 56, 91, 92, 93]
	≤30 days	[94–96, 97, 45, 98, 65, 99, 100, 95, 101, 94, 102, 103, 104, 93]
	<1 year	[45, 72, 74, 95, 96, 105, 106]
	≥1 year	[10, 77, 87, 96, 107]
Phenotype/Classification	Unsupervised learning	[108–114]
	Semi-supervised learning	[115]
	Supervised learning	[116, 117]

In the context of ML applications for predicting readmissions, phenotyping and mortality in HF patients, several key pre-processing steps and evaluation techniques are essential for developing robust predictive models. Based on the findings of Naser et al. [118], Table 2 summarizes the preprocessing steps and applied evaluation metrics of ML algorithms. It has been supplemented by relevant findings from the individual studies and supported by literature sources. The preprocessing phase includes several critical steps to ensure data quality and suitability for analysis. One of these steps is data cleaning, which is performed to address issues such as missing values, duplicate entries, null values and outliers. This step is crucial for improving the integrity of the dataset [55, 73, 77]. Once the data are cleaned, data transformation is performed to convert categorical values to numerical values, normalize data distributions, and correct incorrect data types [72, 86].

**Table 2:** Examples of applied pre-processing steps and applied evaluation metrics of ML algorithms

	Category	Possible Steps and applied Metrics	Source example
**Preprocessing steps**	Data cleaning	Handling and removing missing values, removing duplicates, cleaning null values, removing outliers,	[55, 73, 77]
	Data transformation	Converting categorical values to numerical values, normalizing data, correcting data types	[72, 86]
	Data balancing	Over-sampling, Under-sampling, using SMOTE	[11, 29, 47]
	Data splitting	Splitting into training data, test data and/or validation data	[52, 119]
	Variable selection	Conducting variable importance ranking	[29]
**Applied Evaluation Metrics of ML models**	Validation	Cross, internal or external validation metrics (in clinical setting or a different dataset)	[26, 51, 73]
	Brier Score	Metrics for assessing calibration of probabilistic predictions	[55, 64, 72]
	Explainability	SHAP, DeepSHAP values	[68, 72, 86]
	Performance metrics	Accuracy, Precision, Recall, F1 Score, AUC-ROC metrics	[30, 42]

Another important aspect of preprocessing is feature scaling, which uses techniques such as min-max scaling and standard scaling to normalize the range of independent variables. In addition, dealing with unbalanced datasets is critical; methods such as over-sampling, under-sampling, and Synthetic Minority Over-sampling Technique (SMOTE) are used to ensure equitable representation of classes, as discussed by Javeed et al. [29], Lin & Zhu [11] and Sakr et al. [47]. Once the preprocessing is complete, the data is commonly partitioned into training, test, and validation sets in preparation for model training.

In the preprocessing step of variable selection, variable importance ranking was applied in several studies to identify the most relevant predictors for model development. Identifying relevant variables through importance ranking is also a crucial step in determining the factors that significantly contribute to predictive outcomes, as highlighted by [29]. Across the included studies, feature selection and importance ranking were reported using methods such as SHapley Additive exPlanations (SHAP) values [68, 72, 86] or Random Forest feature importance [111], where available. However, reporting of these methods was inconsistent across studies, and several studies did not explicitly describe their feature selection approach. This limits comparability and synthesis of variable importance across models. Details on the most relevant variables for prediction of mortality and readmission outcomes are provided in Appendix 1 and Appendix 2.

The next phase involves the application of various ML algorithms tailored to the objectives of the study. After model training, rigorous evaluation of the ML models is required to assess their performance and reliability. Validation processes can be cross, internal or external, performed within the available dataset, in a clinical setting or using a different dataset to ensure that the models are able to generalize well beyond the training data [52, 119]. To assess model performance, metrics such as the Brier Score are used to measure the calibration of probabilistic predictions. This metric assesses how confident the model is in its predictions, as elaborated by Austin et al. [64], Chen et al. [72] and Tanaka et al. [55]. In addition, model explainability is a critical consideration, with techniques such as SHAP and DeepSHAP used to demonstrate the interpretability of ML algorithms, allowing stakeholders to understand the decision-making processes [68, 72, 86]. In addition, a comprehensive evaluation of performance metrics such as accuracy and Area Under the Curve (AUC) is performed to determine the effectiveness of the models in accurately predicting outcomes. These metrics provide a holistic view of model performance and help inform the decision-making process regarding their use in clinical practice [30, 42].

### Prediction of mortality

In the analysis of the literature, 68 relevant studies were assigned to the outcome of mortality. Due to the close relationship, the studies that examined survival in HF patients were also included in the mortality group. As shown in Appendix 1, the AI models used were able to make predictions for the next 2 days up to 6 years, depending on the dataset. The most commonly used public datasets are the Medical Information Mart for Intensive Care (MIMIC-III) that contains the identified health data associated with patients admitted to critical care units and those datasets stored on UCI. Most studies indicate how many variables they ultimately import into the AI models, as the variables are sorted by relevance using various techniques and included in further studies accordingly. There are also studies that do not explain exactly how many of the available variables were finally included in the AI model. If the studies did not explicitly mention the final selection of the variables, the original number of variables was included in Appendix 1. The study with the lowest number of variables (4) was by Misumi et al. [40] and the study with the highest number of variables (1222) was by [120].

After comparing different ML algorithms in the studies, 21/68 studies recommended Random Forest (RF) and 7/68 recommended XGBoost. Both are based on decision trees, robust to overfitting and allow the importance of individual variables to be determined. Across all studies, the variables age with 30 mentions, serum creatinine level with 21 mentions, serum sodium level/concentration with 16 mentions, ejection fraction (EF) with 13 mentions, systolic blood pressure as well as platelets with 12 mentions each and blood urea nitrogen (BUN) with 10 mentions are among the most frequently mentioned relevant variables for predicting mortality. Other relevant variables with more than 5 mentions are NT-proBNP/BNP with 9 mentions, diastolic blood pressure with 8 mentions, sex with 8 mentions, BMI with 7 mentions, and haemoglobin with 6 mentions. The highest reported accuracy and AUC score among all studies was achieved by Özbay Karakuş & Er [73] using support vector machines (SVM), with both scoring 1.0. It is critical to note that although these perfect scores of 1.0 indicate an extremely effective model, it is essential to rule out overfitting and therefore conduct a more thorough examination of the data. Additionally, it can be observed that a significant number of studies did not report AUC values which reduces comparability and completeness. Furthermore, some studies lacked the accuracy score or did not document relevant variables.

### Prediction of readmission

A total of 32 studies were identified that investigated the outcome of ‘readmission’ after a diagnosis of HF using different ML techniques (see table in Appendix 2). The majority of studies employed a variety of data sources, including EHR systems from different hospitals, to obtain a comprehensive range of results pertaining to readmissions of patients with HF. Only a few data sources were used by two studies each, such as the MIMIC-III database system, the TOPCAT study, the Hospital Morbidity Data Collection (HMDC) or Physionet. Consequently, the number of variables differs considerably, with the data sources in a total of 19 studies containing between 9 and 146 variables.

The most frequently employed modelling techniques for predicting readmission encompass RF (18 studies), SVM (15 studies), Gradient Boosting Machine (10 studies), XGBoost (9 studies), and K-Nearest Neighbors and Decision Trees (8 studies each). Although logistic regression is not a specific ML technique, it was employed as a benchmark for other ML techniques in nearly 14 studies. However, other machine learning techniques, including AdaBoost, neural networks and CatBoost, were also employed as comparative techniques. In seven studies, the RF was identified by the authors as the proposed algorithm, as it demonstrated the most accurate results in comparison to other machine learning techniques for the prediction of resumption. The accuracy values ranged from 0.574 to 0.940, with Burugadda et al. [91] achieving the latter value with an AUC of 0.98, representing the highest value observed in all studies examining the prediction of readmission.

Across all studies, pre-existing comorbidities with 9 mentions, BUN and age with 8 mentions each were identified as the most relevant variables for the readmission of patients with HF. Furthermore, the analysis revealed that CHF type, previous hospitalizations and hemoglobin are also relevant variables for readmission, with six studies each identifying these as significant factors. With regard to RF, the most relevant variables, namely BUN and age, are also confirmed with three mentions each from a total of seven studies. Other variables that were identified as relevant following the RF were the ejection fraction, Kansas City Cardiomyopathy Questionnaire (KCCQ) score and the glomerular filtration rate, which were each mentioned twice.

It is essential that the results of the studies consider the general prediction of readmission over different periods, including 30 days, 90 days, 100 days, 6 months, 1 year, 3 years and even 6 years. This could result in disparate outcomes for the ML techniques employed or compromise the comparability of the values. It is also noteworthy that 15 studies did not present any accuracy values for readmission, thereby limiting the possibility of conducting a comprehensive comparison. Furthermore, the comparability of the relevant variables is also constrained, as the studies in question specify disparate numbers of variables that are pivotal for predicting readmission. Of these, four studies did not provide any information regarding the most salient variables.

### Prediction of phenotypes and classes

In the recent literature, 10 studies have applied phenotyping and clustering techniques to patient data to improve the understanding and management of HF. It is noticeable that the majority of studies deal with the outcomes mortality and readmission, (e.g. [111–113]), one study only mortality [117] and one study classification of HF with preserved ejection fraction (HFpEF) and HF with reduced Ejection Fraction (HFrEF) [115]. Others have distinguished between HF and non-HF groups [27]. More detailed classifications include phenotypes based on mortality risk (low, intermediate, high) [117] or specific patient profiles including clinical characteristics such as age, renal function, gender, comorbidities and biomarkers [108, 112, 114]. Some studies also examined phenotypes associated with frailty and biomarker levels [111] or classified patients into NYHA classes [116]. Further classifications included biomarker differences and clinical phenogroups [109, 113] or symptoms such as dyspnea and indigestion [110]. The results show that at least 2 clusters, such as Dixit & Chattopadhyay [115]and Stienen et al. [113], and a maximum of 6 clusters, such as Urban et al. [114] and Gevaert et al. [109], could be identified by the ML techniques used. The size of the datasets varied widely, from a minimum of 10 variables to a maximum of 349 variables analyzed, and the majority used data from the medical records of the hospitals involved rather than public datasets. Three studies provided information on the accuracy of the proposed algorithms, which ranged from 0.8887 to 0.9954. Among the unsupervised learning studies, k-means clustering and hierarchical clustering were the most commonly used techniques. In 5 studies, information could be found on relevant variables that influence clustering or differ significantly between patient clusters. The variable BNP/NT-proBNP was mentioned in 5 studies, age in 4 studies, creatinine in 3 studies, atrial fibrillation in 2 studies and haemoglobin in 2 studies.

### Other outcomes

In addition to the outcomes of mortality, readmission, and phenotypes, further outcomes have been investigated in the literature that can be categorized as other outcomes. A total of 10 studies can be identified in this regard. These include the prediction of costs for HF patients, which arise from hospitalization costs, outpatient costs, and medication costs [77, 121]. Another outcome consists of worsening HF events, which are considered a critical indicator of disease progression in HF patients [55, 96, 122, 123]. Early analysis of these events facilitates the development of predictive models that promote early intervention. Another application area of ML techniques is the prediction of HF patients’ need for advanced therapies and their transfer to the Cardiac Intensive Care Unit [60, 124]. Furthermore, Guidi et al. [125] developed a score-based prediction as well as chronological follow-up comparisons and the assessment of HF severity. Balabaeva et al. [126] investigated the prediction of CHF episodes along with heart rate value prediction.

## Discussion

With the growing number of AI studies analyzing data from chronic HF patients and making predictions about outcomes such as mortality risk, hospital readmissions, phenotypes and other outcomes, reviews help to visualize and consolidate research progress. The present research uses a literature review to show not only which ML algorithms best predict mortality in HF, but also readmission in hospitals and phenotyping of HF patients. Our findings from our review of the most relevant variables are consistent with previous studies, such as the one by Yu & Son [127]. Additionally, Yu & Son [127] further categorized the significant predictors into groups such as socio-demographics, vital signs, medical history, therapy, medication and laboratory results.

With regard to RQ2, various implications for practice and research can be derived from this study. Physicians should consider the relevant variables identified in this review to detect changes in HF and make informed treatment decisions. In relation to RQ1, it can be stated that age is a relevant factor in mortality and readmission. In the case of mortality, there is a consensus on creatinine, sodium, platelets and EF as being the most relevant to the AI algorithms in the studies. The mortality studies are the most represented and cover a wide range of time periods studied. Therefore, the information on the relevant variables is most represented here. Other relevant variables include systolic and diastolic blood pressure, BUN, NT-proBNP/BNP, sex and BMI. The mortality and readmission groups have two variables in common that were frequently mentioned: BUN and hemoglobin. The readmission group is the second largest group in our review, with comorbidities, CHF type and previous hospitalizations being the most frequently mentioned variables. Thus, these variables should be prioritized in telemonitoring platforms. Clinical dashboards should flag significant derivations in these variables and support automated alerts for clinicians, potentially enabling earlier interventions. Table 3 synthesizes the key findings from RQ1 and systematically maps them to their corresponding implications for research and practice in addressing RQ2. **Concerning clinical decision-making, high-yield predictive variables should be integrated into clinical monitoring systems to enable earlier interventions (I1).** The comparison between influencing variables of mortality and readmission should serve as an overview for future AI studies to predict HF deterioration. **Selecting and prioritizing consolidated variables from existing evidence can improve the robustness and relevance of AI models in predicting HF outcomes (I2).** In this way, researchers can check in advance whether the consolidated relevant variables are available in their data concept for AI model development. In the case of a prospective study, data can be collected from patients in clinics or from suitable wearables. This approach can lead to improved and simplified clinical decision making. The number of variables used in AI algorithms varies widely. On average, 117 variables were examined in the studies analyzed. As can be seen from the individual studies, not all variables have the same influence on the predictions of the AI algorithms. For example, age has one of the strongest influences on mortality according to Newaz et al. [42], but not in the study conducted by Zamen [128], although both use the same dataset and propose RF as the best algorithm.

**Table 3:** Structured mapping of key findings from RQ1 to implications for research and practice addressing RQ2

RQ1 key finding	What this means	RQ2 implication	Linked implication
Age and other recurrent predictors were identified across mortality and readmission.	Core predictors should inform risk monitoring.	**Integrate** key predictive variables into monitoring systems and telemedicine tools.	I1, I4
Mortality/readmission models depend on predictor selection: Mortality was mainly linked to clinical (e.g., ejection fraction, blood pressure) and laboratory markers (e.g., creatinine, sodium, platelets, BUN, NT-proBNP/BNP), whereas readmission was more often associated with comorbidities, CHF type, prior hospitalizations, BUN, and hemoglobin.	Variable selection should be evidence-based.	**Prioritize** evidence-based predictors and **validate** them externally.	I2, I5
Phenotyping findings are heterogeneous and rarely translated into care.	Phenotyping should move beyond classification.	**Use** AI-based phenotyping to support personalized care.	I3
Dataset limitations and heterogeneity undermine the robustness of predictors and AI models	Better infrastructure is needed.	**Build** larger datasets and **standardize** data collection and validation.	I7, I8, I9
Limited explainability reduces trust in model predictors and limits clinical uptake	Transparency is needed for implementation.	**Embed** explainability to strengthen trust and reliability.	I6

Based on the phenotypes identified, few matches could be found between the 10 hits, as the databases are individual, e.g. underlying data, number of patients and study objectives are different. Nevertheless, these studies demonstrate the broad applicability of AI to large datasets. Reviewing the identified phenotypes and classifying the patients helps to understand the large datasets and provides a better understanding of the chronic disease. **Phenotype studies should move beyond classification to provide actionable recommendations for personalized therapy or quality-of-life improvements (I3).** In the future, phenotype studies should go a step further and recommend appropriate therapy or quality of life recommendations based on the phenotype, as in related medical fields. During our search, we categorized some studies as other outcomes because they did not fit the study focus but were worth mentioning. Some of these were dealing with predicting costs in people with HF. These are particularly useful for future cost-benefit analyses of the use of AI in chronic diseases such as HF. With our review, we have shown which type of AI has been used to predict mortality or readmission, which variables are best suited for prediction, and what the performance and data inputs were. The development of such AI models requires time, human and financial resources. Therefore, future research should continue to evaluate how much money AI can save in everyday medical practice and how it can contribute to better patient care.

Software and hardware developers in the field of telemedicine should incorporate the relevant variables into applications and tools, such as trackers and sensors. **Incorporating evidence-based variables into telemedicine tools can enhance the precision of early detection and enable proactive treatment strategies (I4).** The use of these sensors can enhance patient education and engagement by encouraging HF patients to become more involved in understanding their condition, thereby allowing them to make more informed health decisions. Early prediction of changes in HF can initiate proactive treatments that potentially reduce hospital admissions and expensive surgical procedures. Monitoring patients through sensors can help streamline clinical tasks and reduce the burden on healthcare personnel, freeing up more resources for direct patient care.

Most studies were retrospective, i.e. they were based on an existing dataset in the clinic or a public database. The datasets are often divided into training, validation and testing. However, it is important to check how the AI models perform on other datasets and whether the researchers conclude with the same ML algorithm recommendation. **Validating AI models with external datasets is critical to ensure generalizability and reliable performance in diverse clinical environments (I5).** Some literature reviews recommend investigating the calibration, external validation, and use of predictive models, convincing clinicians to use these predictive models, and increasing the transparency of AI models so that it is possible to understand how the AI model works [4, 12]. Olsen et al. [7] recommend that the algorithms should be validated by external cohorts, made broadly applicable to different hospital systems, continuously monitored, and designed to be cost-effective to develop and implement in order for them to be used in clinical practice. The generalizability and applicability of the trained algorithms is still lacking. Future studies should validate their results with external cohorts, ideally in a clinical setting. With regard to explainability, various techniques are used to indicate the influence of individual variables on the AI models. However, this information is not provided in all studies, so a conclusive comparison of the relevant parameters is not possible. Therefore, this review can only provide a selection of relevant parameters for the prediction of readmission and mortality. **Explainability should be integrated as a standard practice in AI model development to enhance transparency and foster collaboration within the research community (I6).** Future AI developers should consider explainability as an important part of development and share the results with the research community. In future applications, SHAP and other explainable AI techniques should be increasingly considered to enhance the transparency and interpretability of AI models, thereby fostering clinical trust, supporting informed decision-making [129].

Many studies in the reviewed literature are based on the same datasets, mostly the UCI dataset, which consists of only 13 variables and 299 patients. This is in line with the research by Azmi et al. [14] and Naser et al. [118], who analyzed ML techniques for the prediction of CDs. To include additional parameters such as ECG values, larger datasets with more variables and patients are needed. **The creation of diverse and large public datasets is essential to improve the accuracy of AI models and support robust predictions in different healthcare settings (I7).** However, caution is needed when interpreting the results and recommending an appropriate AI algorithm. The majority of studies do not use 100% of the data provided because the datasets are not complete. The variables with missing proportions are either not included in the AI development or are artificially imputed using various techniques. These limitations also apply to this literature review. **Incomplete datasets and imputation techniques highlight the need for comprehensive, high-quality data to enhance the reliability of AI model outcomes (I8).** In this aspect and with regard to effective clinical decision-making, national or international efforts should be made to standardize telemonitoring data collections and models should be trained and validated on harmonized datasets that reflect the target population, i.e. HF patients. **Efforts are necessary in order to standardize the telemonitoring data collection and models should be trained and validated reflecting the target population (I9**). The AI algorithms can make more reliable predictions if they are trained, tested and validated on real data. For further research, complete datasets with different variables from laboratory, clinic, demographic, economic, EHR, etc. are needed. These should cover a large period over several years, be suitable for outcomes such as readmission, mortality and phenotyping, and be made available to AI developers via a public database.

As every research study has its limitations to a certain extent, so does this study. Only publications from the Scopus database were included in the literature review. Additional databases such as Web of Science, PubMed, etc., or a search of specific medical informatics journals might have provided even more relevant records. Including additional databases, such as PubMed or IEEE Xplore, could enhance the comprehensiveness of our review. However, we chose Scopus due to its broad interdisciplinary coverage and significant overlap with other databases, such as PubMed. A forward and backward search would have identified further potentially relevant sources and extended the findings. Although the approach followed related work and methodological guidelines, selection bias among researchers cannot be completely ruled out. Often, only studies with significant results are published, in this case predominantly those with high accuracy and AUC scores for the ML techniques used. Studies with negative or non-significant results are less likely to be published, which can skew the representation of the actual research landscape. Moreover, not all included studies have complete data fields. Often, accuracy scores, AUC values, relevant variables statements, or test splits are missing. With regard to feature selection methods, it should be noted that the specific use of feature selection methods like SHAP or Least Absolute Shrinkage and Selection Operator (LASSO) can influence the identification of key variables concerning mortality, readmission and phenotyping. SHAP tends to explain individual predictions LASSO, on the other hand, selects features based on their contribution to model accuracy or loss reduction, but it may overlook weak yet important features.

The contributions of this study are twofold. Firstly, it is, to the best of the authors’ knowledge, the first review to address the potential HF outcomes mortality and readmission as well as phenotyping. Thus, it contributes to scientific research by closing this research gap. Secondly, practitioners can use the results of this literature review by incorporating the results of the ML analyses into their treatment decisions.

## Conclusion

To answer RQ1, information on the relevant variables was extracted from the studies as part of the systematic literature review. The review of the full texts showed that it was useful to group the studies into outcomes of readmission, mortality and phenotyping. This made it possible to highlight the relevant variables in each group. In addition, the different predictive capabilities of each outcome were identified, which can vary from two days to 6 years. To answer RQ2, the different techniques for preparing the dataset for AI development were considered and summarized. In addition, relevant recommendations for research and practice were derived from the findings. The review also provided further recommendations for future research to improve the use and evaluation of AI algorithms in HF.

## Electronic supplementary material

Below is the link to the electronic supplementary material.

Supplementary material 1

Supplementary material 2

Supplementary material 3

Supplementary material 4

Supplementary material 5

## Data Availability

Not applicable.

## Abbreviation

HF: Heart Failure
AI: Artificial Intelligence
AUC: Area Under the Curve
BUN: Blood urea nitrogen
CD: Cardiovascular disease
ECG: Electrocardiogram
EF: Ejection fraction
HER: Electronic health record
EPR: Electronic patient records
EU: European Union
HF: Heart failure
HFpEF: Heart failure with preserved ejection fraction
HFrEF: Heart Failure with reduced Ejection Fraction
HMDC: Hospital Morbidity Data Collection
ML: Machine learning
NYHA: New York Heart Association
RF: Random Forest
SHAP: SHapley Additive exPlanations
SMOTE: Synthetic Minority Over-sampling Technique
SVM: Support vector machine
