# Supplementary material for: A systematic review of machine learning algorithms for mortality risk, readmission and phenotype prediction in patients with heart failure: exploring key data sources, input variables and outcomes
Source: BMC Med Inform Decis Mak. 2026 Jun 3;26:205. doi: 10.1186/s12911-026-03560-8 (PMC13235149; doi:10.1186/s12911-026-03560-8)
Supplement: Supplementary file 1 — Supplementary material 1 [file 12911_2026_3560_MOESM1_ESM.docx]

**Appendix 1:** **Research results on mortality**

| Source | Considered ALG | Proposed ALG | Accuracy | AUC | Data source | No. of variables | Most relevant variables | |
| --- | --- | --- | --- | --- | --- | --- | --- | --- |
| Austin et al. (2022) | LOG-REG, NN,RF, XGBoost, LL-REG | LOG-REG | na | 0.794*, 0,755** | EHMRG, EFFECT Phase II | 111 | time to event*, SNa, long-term care residence status** | |
| Javeed et al. (2023) | NB, LR, DT, RF, KNN, AdaBoost, SVM | RF* | 0.9459* | 0.94* | EHR Data | 10 | na | |
| Tanaka et al. (2024) | CART, RF, XGBoost | XGBoost | na | 0.816 | KCHF registry | na | SOD, BUN, AGE | |
| Sakr et al. (2017) | DT, SVM, ANN, BC, BN, KNN, RF | RF | na | 0.97* | FIT registry | 49 | AGE, METS, HR, HX HET, SBP, HX Diabetes, DIU, Afib, Other HET Med, Diabetes Med, Family HX CHD, HX SMO, RACE, SEX | |
| Desai et al. (2020) | LASSO-REG, CART, RF, GBM | GBM | na | na | EMR | 54 | *Frailty score, AGE, POV, PH, BUN, SNa, SC, LVEF, BNP | |
| Xu et al. (2024) | LOG-REG, SVM, BN, RF, XGBoost, Bi-LSTM (RNN), Retain (RNN), Dipole (Bi-RNN), Adacare, SAnD, HiTANet, PC-HMM, IoHAN, DAJLENet (NN) | DAJLENet (NN) | 0.835*, 0.825** | 0.812*, 0.810** | MIMIC-III database | na | Aortic aneurysm, Unspecified essential hypertension, AKF, CKD, Stage IV (severe), (S) SEPS, CHF, Extracorporeal circulat, ARF*** | |
| McGilvray et al. (2022) | DL | DL | na | 0.91 | EHR Data | na | SBP, BNP, LOS | |
| Xu et al. (2023) | LOG-REG, NB, RF, KNN, DT, XGBoost | RF | 0.7896 | na | EMR | 45 | *PH, AGE, GFR, BNP, NYHA Class, TLC, SALB, HB, CHOL, PASP **PH, AGE, GFR, BNP, DBP, SBP, NYHA Class, SUA, | |
| Chen at al. (2024) | RF, SVM, XGBoost, LOG-REG | SVM | na | 0.948* | Medical records | na | BUN, CD4-T, Neutrophil, NT-proBNP, AGE, DBP, eGFR, SBP, CREA, NYHA Class | |
| Lin et al. (2023) | XGBoost, LightGBM, DFS-enhanced LightGBM | DFS-enhanced LightGBM | 0.9508 | na | MIMIC-III database | 48 | UO, PH, Anion gap, Leucocyte, temperature, blood Na, gender | |
| Zhao et al. (2022) | RF, LASSO COX-REG, LOG-REG, R-COX, GBDT, E COX-REG, SVM | LASSO COX-REG | na | 0.78*, 0.75** | TOPCAT dataset | 72 | *AGE, RACE, stroke, Diabetes  ** RACE, CHF-HOSP, COPD, SMOK, BG | |
| Angraal et al. (2020) | RF, SVM, LL-REG, GDB, LOG-REG | RF | na | 0.72 | TOPCAT dataset | 86 | BUN, BMI, KCCQ, APL, AGE | |
| Sabouri et al. (2023) | RF, SVM, KNN, XGBoost, LOG-REG, MLP, NB, QDA | LOG-REG (RFE) / LOG-REG (Boruta) | 0.84 / 0.85 | 0.91 / 0.90 | Clinical data | 34 | *Inotrop (drug), RD, Abnormal LFT, Dialysis, Edema, SCREA, eLVEF, AGE  ** CKF, SBP, UA | |
| König et al. (2021) | GLM, RF, GBM, XGBoost, sNN | GBM / XGBoost | na | 0.882/ 0.882 | administrative routine data | na | na | |
| Tian et al. (2023) | RF, SVM, XGBoost | XGBoost | 0.854* | 0.916 | Clinical data | 67 | AGE, ALT, AST, SNa, SK, NT-proBNP, HB, RBD, etc. | |
| Jing et al. (2020) | RF, XGBoost, LOG-REG | XGBoost | na | 0.77 | Geisinger EHR Data | 209 | na | |
| Huang et al. (2024) | DNN, LOG-REG, SVM, RF | DNN | 0.874 | na | Clinical data | 20 | HF score, PCI, AGE, NT-proBNP, Na, HB | |
| Ketata et al. (2023) | RF, SVM, XGBoost, LOG-REG | RF | 0.833 | na | UCI | 13 | EF, SCREA, AGE, SNa, Platelets, CPK, BP, AN, SEX, SMOK, Diabetes | |
| König et al. (2022) | GLM, RF, GBM, NNET, XGBoost | XGBoost | na | 0.856 | administrative routine data | na | na | |
| Espinoza et al. (2024) | RF, SVM, DT | RF | 0.92 | na | medical records | 13 | na | |
| Tohyama et al. (2021) | RF, SVM, LL-REG, GBT, NN, voting classifier | SMART-HF | 0.712 | na | JROADHF | 89 | BI, AGE, BMI, LOS | |
| Segar et al. (2022) | LOG-REG, ML |  | na | na | AHA GWTG-HF registry | 65 | *BUN, AGE, BNP, SBP, TRO, DBP, income, RF, Na, vacancy rate, HB, CREA, K, HDL, unemployment rate, etc. ** AGE, BUN, BNP, SBP, TRO, DBP, RF, Na, FPG, Abnormal ECG morphology, CREA, K, etc. | |
| Awan et al. (2019) | RF, wRF, SVM, wSVM, DT, LOG-REG, MLP, | MLP | 0.6493 | 0.628 | HMDC | 47 | na | |
| Wang et al. (2018) | FRNN, DRM, FRkNN, GFRNN, kNN | DRM | na | 0.873*, 0.8845**, 0.8484*** | clinical data | 482 / 618 / 527 | na | |
| Lv et al. (2021) | RF, SVM, ANN, XGBoost, LOG-REG | na | 0.83-0.97 | 0.91-1.00 | EHR Data | 79 | * BUN, hs-cTnI, AST, PLYM ** hs-cTnI, BUN, RF, PBAS, PNEU | |
| Sarijaloo et al. (2021) | LOG-REG, LASSO, GBM, RF, SVM, Combined LASSO + LOG-REG | Combined LASSO + LOG-REG | na | 0.760 | EPIC EHR | 98 | ALB, NT-proBNP, HCO3, Na | |
| Park et al. (2022) | B-COX | B-COX | na | na | STRATS-AHF registry | 27 | *AGE, RD, NP, SNa, SEX, DBP, RAS inhibitors, LV GLS, BMI | |
| Li et al. (2022) | RF, SVM, XGBoost, LOG-REG | XGBoost | 0.826 | 0.824 | eICU-CRD | na | BUN, AGE, average noninvasive BP, UO, Max. RR, min. noninvasive BP, WBC, O2 Satuation | |
| Misumi et al. (2023) | LASSO-REG (4V-RS) | LASSO-REG (4V-RS) |  | 0.783 | REALITY-AHF, NARA-HF | 4 | BP, BUN, SCI, CRP | |
| Gao et al. (2024) | mDL |  |  | 0.838? | MIMIC-III, MIMIC-IV v1.0, eICU v1.2 | 52 | shapley; mental status, GCS, UO, mechanical ventilation, activity, RR. | |
| Stampehl et al. (2020) | LOG-REG, CART, sLOG-REG | sLOG-REG | na | na | Clinical data | 18-34 | *SEX, DICF, DSNF, LDIU, morphine, CCI, Thoracentesis, malnutrition, non-invasive ventilation, tranfusion of packed cells, pulmonary hypertension **SEX, DICF, DSNF, LOS, morphine, CCI, dementia, malnutrition, non-invasive ventilation, tranfusion of packed cells, PNA | |
| Guo et al. (2020) | RF, LOG-REG, DNN | DNN | na | 0.82 | Synthetic EHR | 27 | *AGE, CREA, BMI, SBP, KF, DBP, SMK, AF age at event | |
| Ali et al. (2024) | LOG-REG, SVM, KNN, DT, SGD, Gboost, XGBoost, MLP | RF | 0.968* | na | MIMIC-III database | 27 | na | |
| Gong et al. (2020) | RAM, DT, SVM, NB | RAM | 0.934 | 0.87 | Medical records | 22 | NYHA, AGE, NT—ProBNP, LVEF, ß-blockers, VAR, high BP, CHD, bronchitis | |
| Negassa et al. (2021) | Super Learner, REG, LOG-REG, LASSO, RF, LOG-REG with variable selection, MARS, Mean, Bayesian Logistic, Boosting, BAG | Super Learner | na | na | EMR | 35 | na | |
| Yang et al. (2022) | CART, RF, SVM, GBDT, LightGBM, Optuna–LightGBM | Optuna–LightGBM | 0.92 | 0.8314 | Medical records | 18 | EF, CREA, BUN, BNP, CAD, TLC, Admission Type, Platelets | |
| Ketabi et al. (2024) | RF, SVM, KNN, DT, XGBoost, LOG-REG, GNB, LDA, GBM, CAT | CAT | 0.81*, 0,.72** | 0.61*, 0.58** | FaRSH Database | 15-25 | *Hb, AGE, PH, BMI, SEX, Na  **family HX of MI, Na, BMI, LOS, SBP, DBP | |
| Sandhu et al. (2022) | BGLM, ANN, CART, SVM, Bag Earth, RF, DT, SVM-GA | SVM-GA | 0.9149 | na | UCI | 13 | (S)CREA, ej | |
| Tong et al. (2023) | COX-REG, RF GBM, LASSO COX-REG | RF | na | 0.811 | EHR | 15-48 | *LVEDD, RBC, UA, BUN, MPV, CHOL, RHF, DD, Na, RDW  **LVEDD, CREA, CHOL, RBC, SBP, UA, MPV, GFR, DD, valsartan tablet | |
| Zhang et al. (2024) | LIN-REG, RF, XGBoost, Bi-LSTM, Diople, Retain, IoHAN, CLA Net | CLA Net | 0.832* 0.826** 0.843*** | 0.808* 0.810**  0.815*** | EHR Data |  | na | |
| Li et al. (2023) | DLS-MSM, SVM, MLP, LOG-REG, RF, LGB, KNN | DLS-MSM | *0.8456, **0.8208, ***0.8856, ****0.7607 | *0.9100, **0.8200, ***0.7500, ****0.7200 | MIMIC-III databases | 90 | *****pCO2, RF, BMI, ARB , Pulmonary circulation disorder (Diagnoses), WBC (Laboratory test), Diuretic (Medicine), Diabetes , CCU stays (ICU information), GLU (Laboratory test), K (Laboratory test), CCB (Medicine), Insert BMI (Indicator vector), Hb, Nitrates (Medicine) | |
| Luo et al. (2022) | GWTG-HF (LOG-REG), SAPS-II, LOG-REG and XGBoost | XGBoost | na | 0.831 | MIMIC-III, eICU-CRD | 24 | Mean anion gap, mean Glasgow Coma scale, UO, mean BUN, max. pO2, AGE, min. glucose, mean calcium, mean RR, mean arterial base excess, CREA, mean temperature, BMI, min. platelet, max. temperature | |
| Bohacik et al. (2015) | BNC, NNC, MLP, OneR, PART, LOG-REG |  | na | na | Hull LifeLab | 10 | na | |
| Sutradhar et al. (2023a) | DT, RF, GB, IBSC | IBSC | *0.90, **0.9275, ***0.9150 | na | Clinical data | 10 | Time, SCREA, EF, AGE, CPK, Platelets, SEX, Diabetes, SMOK | |
| Sarswat et al. (2019) | RF, DT, LOG-REG | RF | 0.8667 | na | UCI | 13 | na | |
| Takahama et al. (2023) | LightGBM, Seattle Heart Failure Model (SHFM), MAGGIC Score | LightGBM | na | 0.8700 | Medical records | 39/15 | *TRO, SBP, BMI, Hematcrit, NT-proBNP, CRP, LDLC, WBC, DBP, CREA, TG, TRPG, BUN, LVEF | |
| Zhang (2024b) | KNN, SVM, DT, RF, XGBoost, GradBoost, Seq2Seq | Seq2Seq model | 0.913 | na | Clinical data | 12 | *AGE, CPK, EF, Platelets, SCREA, SNa, Time | |
| Chiu et al. (2022) | RF, SVC, KNN, LightGBM, Bagging, Adaboost, Stacking | Stacking | *0.9525, **0.8690, ***0.8564, ****0.8551 | na | MIMIC-III database | 16 | *Platelets, BG, BUN, AGE, HR, SBP, WBC, mean BP, DBP, prothrombin time | |
| Kwon et al. (2019) | DL, RF, LOG-REG, SVM, BN, GWTG-HF score | DL | na | *0.880, **0.782,  ***0.813 | EHR | na | na | |
| Somavilla et al. (2022) | KNN, SVM, RF, LOG-REG, Polynomial regression, Multivariate regression | Polynomial regression | *0.94 |  | UCI | 10 | AGE, EF, SCREA, high BP, Na, CPK | |
| Sutradhar et al. (2023b) | DT, GB, SVM, ET, CBCEC | CBCEC | 0.9367 | 0.98 | Clinical data | 10 | * time, SCREA, EF, AGE, cr_ph, Plateletes, SNa, SEX ** time, SCREA, EF, AGE, AN | |
| Panyamit et al. (2022) | KNN, LOG-REG, NB, SVM | CART | 0.948 | na | UCI | 13 | CPK, Platelets, Time |  |
| Ishaq et al. (2021) | AdaBoost, DT, ETC, GBM, LOG-REG, NB, RF, SGD, SVM | ETC | 0.9262 | na | UCI | 13 | Age, CPK, EF, Platelets, SC, (S)Na, Time |  |
| Mamun et al. (2022) | DT, EL, GBM, KNN, LOG-REG, RF, SVM | GBM | 0.85 | 0.93 | UCI | 13 | na |  |
| Moreno-Sánchez (2023) | AdaBoost, CART, CPH, GBM, RF, SVM, XGBoost | GBM | 0.74 | na | UCI | 13 | na |  |
| Ay et al. (2023) | KNN, LOG-REG, NB, RF, SVM | KNN | na | 0.85 | UCI | 20 | na |  |
| Saqlain et al. (2016) | DT, LOG-REG, NB, NN, RF, SVM | NB | 0.867 | 0.924 | AFIC | 30 | na |  |
| Kumar et al. (2024) | AdaBoost, CART, DT, GBM, GM, KNN, MLP, NB, RF, SVM | NB | na | na | AIIMS | 33 | Diabetes, Gender, HR, (S)Na, SBP |  |
| Umer et al. (2022) | BN, NN | NN | 0.9263 | na | UCI | 13 | na |  |
| Ahmad et al. (2018) | RF | RF | 0.83 | na | Swedish HF Registry | 86 | na |  |
| Ali et al. (2023) | DT, GBM, RF, XGBoost | RF | 0.9778 | na | Kaggle | na | Age, CPK, EF, Platelets, SC, (S) NA |  |
| Qadri et al. (2024) | BGM, DT, KNN, LOG-REG, NB, RF, SVM, XGBoost | RF | 0.97 | na | UCI | 13 | na |  |
| Zaman et al. (2021) | DT, RF, XGBoost | RF | 0.9998 | 0.99 | UCI | 13 | EF, (S) NA, Time |  |
| Newaz et al. (2021) | AdaBoost, KNN, LOG-REG, RF, SVM | RF | 0.7293 | na | UCI | 11 | Age, EF, SC |  |
| Fozilijonova and Wasito (2022) | DT, KNN, RF | RF | na | na | UCI | 13 | na |  |
| Barfungpa et al. (2024) | SMOTEHDL | SMOTEHDL | 0.9552 | na | UCI | 13 | na |  |
| Özbay Karakus and Er (2022) | LOG-REG, NB, Linear SVM, Cubic SVM , Q SVM, ANN, FG-SVM, KNN, BOT, BAT, FT, MT, CT , RUSB Trees, SubD, mNN | SVM | 1.0 | 1.0 | UCI | 13 | na |  |
| Sachdeva et al. (2023) | DT, RF, SVM, XGBoost | SVM | 0.9667 | na | UCI | 12 | na |  |
| Tsehay et al. (2024) | DT, KNN, NN, REG, XGBoost | XGBoost | 0.865 | 0.93 | Medical records | 10 | Age, Anemia, EF, Platelets, SC, (S)Na, Time |  |
| Zhou et al. (2021) | GA-KPLS, LASSO-REG, LOG-REG, RF, RR, SVM | SVM, GA-KPLS, RR | 0.968, 0.853 | 0.955, 0.734 | FHS | 11 | na |  |
| Sannino et al. (2021) | SVM, NB, ANN, LR, RF, DEREx | DEREx | 0.785 | na | UCI | 10 | CPK, EF, SC |  |
| **Legend**: ALG= Algorithm;  **Considered ALG**: ANN=Artificial Neural Networks; BAG=Bagging; BGM=BGM Classifier (Bayesian Gaussian Mixture); COX-REG=Cox Regression; DL=Deep learning; DT=Decision Tree; F KNN=Fine KNN; GB=Gradient Boost; GLM=Generalized linear models; GNB=Gaussian Naïve Bayes; KNN=K-Nearest Neighbor; LASSO-REG=Lasso Regression; LL-REG=Logistic Lasso Regression; LOG-REG=Logistic Regression; NB=Naïve Bayes; NN=Neural Network; RF=Random Forest; SVM=support vector machine;  XGBoost=XGBoost  **Most relevant variables**: (A)KF=(Acute) kidney failure; (A)RF=(Acute) Respiratory failure; (e)GFR=(estimated) Glomerular filtration rate; (e)LVEF=(estimated) Left Ventricular Ejection Fraction; (S) SEPS=Severe sepsis with septic shock; (S)ALB=(serum) Albumin; (S)CI=(serum) chloride level laboratory tests; (S)CREA=(Serum) Creatinine; (S)K=(serum) potassium; (S)Na=(Serum) Sodium / sodium concentration; (S)UA=(Serum) Uric acid; (V)AR=(ventricular) Arrhythmia; Afib=Atrial Fibrillation; AH=Asphyxia (and hypoxemia); AHF=Acute HF; AIA=antineoplastic and immunomodulating agents; ALT=alanine transaminase; AMI=acute myocardial infarction; AN=Anemia; APL=alkaline phosphatase level; ARB=Angiotensin II Receptor Blocker; ASP=Aspirin use; AST=creatinine aspartate aminotransferase; AT=Arthritis; BA=Bronchial; asthma / Asthma bronchiale; BB=Beta-blocker use; BCL=Extremes of bicarbonate levels; BG=blood glucose; BI=Barthel index for Activities of Daily Living; BMI=body mass index; BS=Blood sugar count; BT=Body temperature; BUN=Blood urea nitrogen level; C30R=Cumulative number of 30-day readmissions; CAD=coronary artery disease;  CCI=Charlson Comorbidity Index; CHD=Coronary Heart Disease; CHF=Congestive heart failure; CHF-HOSP=previous hospitalization for cardiac heart failure; CHOL=Cholesterol; CKD=chronic kidney disease; COPD=chronic obstructive pulmonary disease; CPK=creatine phosphokinase; CRF=chronic renal failure; CRP=C-reactive protein test; CV D=Cardiovascular diagnoses; CV Med=Cardiovascular medication use; DBP=diastolic blood pressure ; DD=Discharge Day; DEP=Depression; Diabetes M=Diabetes (Mellitus); Dialysis=Dialysis; DICF=discharge to intermediate care facility; DIU=Diuretic use; DL=Dyslipidemia; DMONTH=Patient’s discharge month; DSNF=discharge to skilled nursing facility; EF=Ejection fraction; EFFECT Phase II=Enhanced Feedback for Effective Cardiac Treatment Phase II; EHMRG=Emergency Heart Failure Mortality Risk Grade; ERD=Initial emergency room diagnosis of heart failure; ERU=Number of emergency room utilization; FEAB=Disorders of fluid/electrolyte/acid-base balance; FPG=fasting plasma glucose; GCS=Glasgow Coma Scale; HB=Hemoglobin levels; HCO3=bicarbonate; HCP=Out-of-hospital visit to allied health professional; HET=Hypertension; HF=Heart failure; HK=Hypokalemia; HMDC=Hospital Morbidity Data Collection; HOT=Hypotension; HR=Heart Rate / Increasing heart rate; hs-cTnI=high-sensitivity cardiac troponin I ; HX=History; ICM=ischemic cardiomyopathy; IHD=Prior ischemic heart disease; ILD=Illicit intake drugs; ITS=Need for inotropic support; IVC=(Dilated) inferior vena cava; K=Levels of blood potassium; KCCQ=Kansas City Cardiomyopathy Questionnaire (KCCQ) subscale scores; LD=Lung disease; LDH=Lactate dehydrogenase concentrations; LDIU=Loop diuretic administration; LDLC=low density lipoprotein cholesterol; LF=liver function test; LOS=Length of hospital stay; LVD=Severe LV dysfunction; LVEDD=Left Ventricular End-Diastolic Dimension; Med=Medication; METS=Metabolic Equivalent; MIMIC-III=Medical Information Mart for Intensive Care III; MPV=Mean platelet volume; NP=Natriuretic peptide; NT=Nitrate use; NT-proBNP=NT-proBNP level / Elevated NT-proBNP; NYHA Class=NYHA cardiac function classification; P=Blood phosphorus; PAP=pulmonary artery pressure; PASP=pulmonary artery systolic pressure; PAY1=Payment method; PBAS=percentage of basophils; PCI=percutaneous coronary intervention; PH=Previous hospitalization / number of hospitalizations; PLYM=percentage of lymphocytes; PM=Pacemaker; PNA=Pneumonia; PNEU=percentage of neutrophils; pO2=Oxygen pressure; POV=Physician office visits; PR=Pulse rate; RAS=RAS inhibitors; RBC=Red blood cell count; RBD=red blood cell distribution; RD=Renal disease; RDW=Coefficient of variation of RDW; RE=Higher number of previous readmissions; RESIDENT=Patient’s local/district of residence; RHF=Right heart failure; RI=Received intervention; RR=respiratory rate; SBP=Systolic blood pressure / Elevated systolic BP; SES=SES index (Socioeconomic Status); SMO=Smoking; TG=Triglycerides; TLC=total lymphocytes count; TLOS=TLOS (Total Length of Stay); TMed=Total number of medications used; TPH=Time since previous hospitalization for heart failure; TR=tricuspid valve regurgitation; TRO=troponin; TRPG=tricuspid peak gradient;  UO=urine output; VC Sign=Echocardiographic sign of overt volume overload; VED=Number of visits to ED; WBC=White blood cell  **Data source:** AHA=American Heart Association; EHR=Electronic Healthcare Record; EMR=electronic medical record; FIT=Henry Ford exercIse Testing; JROADHF=Japanese Registry of Acute Decompensated Heart Failure; KCHF=Kyoto Congestive Heart Failure registry; UCI=University of California at Irvine repository" | | | | | | | |  |
