# Supplementary material for: A systematic review of machine learning algorithms for mortality risk, readmission and phenotype prediction in patients with heart failure: exploring key data sources, input variables and outcomes
Source: BMC Med Inform Decis Mak. 2026 Jun 3;26:205. doi: 10.1186/s12911-026-03560-8 (PMC13235149; doi:10.1186/s12911-026-03560-8)
Supplement: Supplementary file 2 — Supplementary material 2 [file 12911_2026_3560_MOESM2_ESM.docx]

**Appendix 2 - Research results on readmission**

| Source | Comparative ALG | Proposed ALG | Accuracy | AUC | Data source | No of variables | Most relevant variables |
| --- | --- | --- | --- | --- | --- | --- | --- |
| Hu and Mo (2024) | DT, RF, LOG-REG, NBC, SVM | NBC | 0.64 | 0.6 | Clinical data | 17 | na |
| Landicho et al. (2020) | NN, RF, LOG-REG, SVM | SVM | 0.610 | 0.602 | NMMC | 90 | BA, AT, HB, BT, ERU, PR, BS, ALB, SEX, HET, ILD, ERD |
| Awan et al. (2019) | DT, RF, LOG-REG, SVM, MLP | MLP | 0.6493 | 0.628 | HMDC | 47 | na |
| Kerexeta et al. (2018) | SVM, NB, wNB | Combined classifiers | na | 0.64 | Clinical data | 17 | na |
| Tong et al. (2023) | MV-COX, LASSO-REG, RSF, GBS | RSF | na | 0.641 | EHR | 146 | LVEDD, RBC, UA, BUN, MPV, CHOL, RHF, DD, SOD, RDW |
| Sharma et al. (2022) | XGBoost, GBM, Adaboost, CatBoost, LightGBM, LSVM, G-NB, RF, DT, LOG-REG, NN | XGBoost | na | 0.654 | administrative health databases | na | HF, COPD, HET, IHD, RD, DEP, PH, HX, HB |
| Soliman et al. (2023) | CatBoost, LSTM | CatBoost | na | 0.68 | Clinical data | 40 | PH, VED, TLOS, LOS, CCI, NT-proBNP |
| Golas et al. (2018) | MO-NN, LOG-REG, GBM, DUN (ANN) | DUN (ANN) | 0.646 | 0.705 | clinical,  operational, financial, and claims data | 3512 | C30R, ARF, SOD, PNA, CI, AKF, Diabetes, RF, FEAB, ALB, AH, AN, HK |
| Zhang et al.(2023) | LOG-REG, RF, LightGBM, GBC, CatBoost | CatBoost | 0.712 | 0.732 | Clinical data | 64 | NT-proBNP, LOS, K, P, TG,  LDH |
| Desai et al.(2020) | LOG-REG, LASSO-REG, CART, RF, GBM | GBM | na | 0.745 | EMR | 62 | HF Type, HOT, TMed, POV, SES index, SK |
| Sarijaloo et al. (2021) | LASSO-REG, RF, GBM, SVM | combined LASSO-REG + LOG-REG | 0.71 | 0.76 | EPIC EHR | 98 | ALB, NT-proBNP, BCL, SOD, Systolic BP, Diastolic BP, HR, LD, CV D, AHF, IVC, VC Sign,  LDIU, LVD, ITS |
| Angraal et al.(2020) | LOG-REG, LL-REG, RF, GD, SVM | RF | na | 0.76 | TOPCAT dataset | 86 | HB, BUN, TPH, KCCQ, GFR |
| Missel et al. (2023) | KNN, GLM, RF, SVM , BAG | BAG | na | 0.766 | Clinical data | 46 | na |
| McKinley et al. (2019) | KNN, rKNN, RF, LASSO-REG, GBM, SVM | KNN | 0.773 | 0.768 | EHR | 29 | RI, AGE, AN, AR, DL, PM, BB, NT, ASP, CV Med |
| Zhao et al.(2022) | RF, LOG-REG, FS-COX, LASSO-REG, R-COX, EN-COX, SVM, GBT | RF | na | 0.78 *  0.84** | TOPCAT dataset | 72 | KCCQ |
| Chicco and Jurman (2020) | REG, RF, TOR, DT, ANN, L-SVM, G-SVM, KNN, NB, GBM | RF | 0.74 | 0.8 | Medical records | 13 | SC, EF, AGE |
| Polo Fritz et al.(2022) | XGBoost, AdaBoost, RF, GBM, (LACE = kein ML) | XGBoost | 0.77 | 0.803 | EHR | 51 | HR, CRP, PH, CCI |
| Lorenzoni et al.(2019) | LOG-REG, GLMN, CART, RF, Adaboost, LB, SVM, NN | GLMN | 0.812 | 0.806 | Clinical data | 14 | AMI, ICM, COPD, Comorbidities |
| Tao et al. (2021) | SVM, DT, COX-REG | SVM | na | 0.910 | Medical records | 22 | obesity, AGE >70 years, treatment with ≥2 antihypertensives, echocardiographic E/e’ ratio >9 and  PAP >35 mm Hg,  DIU, spironolactones, diabetes, anaemia, thyroid dysfunction, tumours, H2FPEF score |
| Pishgar et al. (2022) | NN, SVM, KNN, DT, RF, XGBoost, CatBoost | NN | 0.841 | 0.930 | EHR; MIMIC-III database | na | severity scores, admission events, demographics, artifcial events, comorbidity events, lab measurement events |
| Chen at al. (2024) | RF, XGBoost, LOG-REG, SVM | SVM | na | 0.948 | Medical records | 35 | BUN, CD4 T, Neutrophil, NT-proBNP, AGE, DBP, (e)GFR, SBP, CREA, NYHA class |
| Burugadda et al. (2023) | ANN, DT, RF, NB, GBM, LOG-REG, SVM | RF | 0.94 | 0.98 | clinical data | na | na |
| Mortazavi et al. (2016) | P-REG, LOG-REG, RF, GBM, SVM | RF | na | 30 day: 0.628 6 month: 0.654 | Clinical data | 472 | BUN, GFR, sex, waist: hip ratio, HX ICM |
| Beecy et al. (2020) | XGBoost-IA, XGBoost-ID, XGBoost-FA | XGBoost-FA, XGBoost-ID | na | 0.756* (XGB-FA), 0.754 (XGB-ID)  0.693** (XGB-FA), 0.701 (XGB-ID) | EHR | 2032  (for XGB-FA) | *discharge to home, serum chemistry values (eg, hemoglobin level, red blood cell distribution width), quantitative ECG variables  **socioeconomic variables (eg, distance from home to a park, total population) |
| Ru et al. (2023) | NN, XGBoost, RF, LOG-REG | XGBoost* RF** XGBoost*** | na | 0.595* 0.630** 0.640*** | US database, Medical claim | na | na |
| Sabouri et al.(2023) | KNN, RF, XGBoost, LOG-REG, NB, SVM, MLP, QDA | Boruta-SVM*  MRMR-KNN** | 0.81*  0.63** | 0.73*  0.6** | Clinical data | 34 | *WRF, Base CREA, Discharge CREA **CRF, PAP, HB, Base CREA, Discharge CREA, TR, IVC, Ascites, Edema, SBP, UA, dialysis, Na, Infection |
| Rahman et al. (2023) | MLP, LDA, RF, XGBoost, LOG-REG, SVM, ET, AdaBoost, KNN, CatBoost, GBM, LightBM, EN-REG |  | 0.8836 (CatBoost)  0.87 (AdaBoost) 0.849 GBM | 0.879 (CatBoost)  0.865 (AdaBoost) 0.861 (GBM) | EHR | 9 | *Admission way, LACE score,  reduced HB, type of heart failure,  occupation, mitral valve EMS,  CCI.score, white globulin ratio |
| Najafi-Vosough et al. (2021) | RF, NB, LS-SVM, SVM, AdaBoost, BAG | RF | 0.91 | na | hospital records | 46 | BUN, ejection fraction, age, Na |
| Liu et al. (2019) | CNN, RF | CNN | 0.757*  0.7188** | na | MIMIC III | na | na |
| Jahangari et al. (2024) | RF, LOG-REG | RF | * 0.633 **0.574 | *0.607 **0.576 | Readmission Database | *31 **30 | * AGE, PAY1, DMONTH, CKD, RESIDENT  ** AGE, PAY1 |
| Rizinde et al. (2023) | MLP, KNN, LR, DT, RF, SVM | RF | na | 0.94 | medical records | 59 | RESIDENT, shortness of breath, max. DBP at rest, max. SBP at rest, max. HR, risk factor of decompensated heart, alcohol intake, sex, number of days for the first hospitalization, AGE |
| **Legend**: ALG= Algorithm;  **Considered ALG**: ANN=Artificial Neural Networks; BAG=Bagging; BGM=BGM Classifier (Bayesian Gaussian Mixture); COX-REG=Cox Regression; DL=Deep learning; DT=Decision Tree; F KNN=Fine KNN; GB=Gradient Boost; GLM=Generalized linear models; GNB=Gaussian Naïve Bayes; KNN=K-Nearest Neighbor; LASSO-REG=Lasso Regression; LL-REG=Logistic Lasso Regression; LOG-REG=Logistic Regression; NB=Naïve Bayes; NN=Neural Network; RF=Random Forest; SVM=support vector machine;  XGBoost=XGBoost  **Most relevant variables**: (A)KF=(Acute) kidney failure; (A)RF=(Acute) Respiratory failure; (e)GFR=(estimated) Glomerular filtration rate; (e)LVEF=(estimated) Left Ventricular Ejection Fraction; (S) SEPS=Severe sepsis with septic shock; (S)ALB=(serum) Albumin; (S)CI=(serum) chloride level laboratory tests; (S)CREA=(Serum) Creatinine; (S)K=(serum) potassium; (S)Na=(Serum) Sodium / sodium concentration; (S)UA=(Serum) Uric acid; (V)AR=(ventricular) Arrhythmia; Afib=Atrial Fibrillation; AH=Asphyxia (and hypoxemia); AHF=Acute HF; AIA=antineoplastic and immunomodulating agents; ALT=alanine transaminase; AMI=acute myocardial infarction; AN=Anemia; APL=alkaline phosphatase level; ARB=Angiotensin II Receptor Blocker; ASP=Aspirin use; AST=creatinine aspartate aminotransferase; AT=Arthritis; BA=Bronchial; asthma / Asthma bronchiale; BB=Beta-blocker use; BCL=Extremes of bicarbonate levels; BG=blood glucose; BI=Barthel index for Activities of Daily Living; BMI=body mass index; BS=Blood sugar count; BT=Body temperature; BUN=Blood urea nitrogen level; C30R=Cumulative number of 30-day readmissions; CAD=coronary artery disease;  CCI=Charlson Comorbidity Index; CHD=Coronary Heart Disease; CHF=Congestive heart failure; CHF-HOSP=previous hospitalization for cardiac heart failure; CHOL=Cholesterol; CKD=chronic kidney disease; COPD=chronic obstructive pulmonary disease; CPK=creatine phosphokinase; CRF=chronic renal failure; CRP=C-reactive protein test; CV D=Cardiovascular diagnoses; CV Med=Cardiovascular medication use; DBP=diastolic blood pressure ; DD=Discharge Day; DEP=Depression; Diabetes M=Diabetes (Mellitus); Dialysis=Dialysis; DICF=discharge to intermediate care facility; DIU=Diuretic use; DL=Dyslipidemia; DMONTH=Patient’s discharge month; DSNF=discharge to skilled nursing facility; EF=Ejection fraction; EFFECT Phase II=Enhanced Feedback for Effective Cardiac Treatment Phase II; EHMRG=Emergency Heart Failure Mortality Risk Grade; ERD=Initial emergency room diagnosis of heart failure; ERU=Number of emergency room utilization; FEAB=Disorders of fluid/electrolyte/acid-base balance; FPG=fasting plasma glucose; GCS=Glasgow Coma Scale; HB=Hemoglobin levels; HCO3=bicarbonate; HCP=Out-of-hospital visit to allied health professional; HET=Hypertension; HF=Heart failure; HK=Hypokalemia; HMDC=Hospital Morbidity Data Collection; HOT=Hypotension; HR=Heart Rate / Increasing heart rate; hs-cTnI=high-sensitivity cardiac troponin I ; HX=History; ICM=ischemic cardiomyopathy; IHD=Prior ischemic heart disease; ILD=Illicit intake drugs; ITS=Need for inotropic support; IVC=(Dilated) inferior vena cava; K=Levels of blood potassium; KCCQ=Kansas City Cardiomyopathy Questionnaire (KCCQ) subscale scores; LD=Lung disease; LDH=Lactate dehydrogenase concentrations; LDIU=Loop diuretic administration; LDLC=low density lipoprotein cholesterol; LF=liver function test; LOS=Length of hospital stay; LVD=Severe LV dysfunction; LVEDD=Left Ventricular End-Diastolic Dimension; Med=Medication; METS=Metabolic Equivalent; MIMIC-III=Medical Information Mart for Intensive Care III; MPV=Mean platelet volume; NP=Natriuretic peptide; NT=Nitrate use; NT-proBNP=NT-proBNP level / Elevated NT-proBNP; NYHA Class=NYHA cardiac function classification; P=Blood phosphorus; PAP=pulmonary artery pressure; PASP=pulmonary artery systolic pressure; PAY1=Payment method; PBAS=percentage of basophils; PCI=percutaneous coronary intervention; PH=Previous hospitalization / number of hospitalizations; PLYM=percentage of lymphocytes; PM=Pacemaker; PNA=Pneumonia; PNEU=percentage of neutrophils; pO2=Oxygen pressure; POV=Physician office visits; PR=Pulse rate; RAS=RAS inhibitors; RBC=Red blood cell count; RBD=red blood cell distribution; RD=Renal disease; RDW=Coefficient of variation of RDW; RE=Higher number of previous readmissions; RESIDENT=Patient’s local/district of residence; RHF=Right heart failure; RI=Received intervention; RR=respiratory rate; SBP=Systolic blood pressure / Elevated systolic BP; SES=SES index (Socioeconomic Status); SMO=Smoking; TG=Triglycerides; TLC=total lymphocytes count; TLOS=TLOS (Total Length of Stay); TMed=Total number of medications used; TPH=Time since previous hospitalization for heart failure; TR=tricuspid valve regurgitation; TRO=troponin; TRPG=tricuspid peak gradient;  UO=urine output; VC Sign=Echocardiographic sign of overt volume overload; VED=Number of visits to ED; WBC=White blood cell  **Data source:** AHA=American Heart Association; EHR=Electronic Healthcare Record; EMR=electronic medical record; FIT=Henry Ford exercIse Testing; JROADHF=Japanese Registry of Acute Decompensated Heart Failure; KCHF=Kyoto Congestive Heart Failure registry; UCI=University of California at Irvine repository" | | | | | | | |
