# Supplementary material for: A systematic review of machine learning algorithms for mortality risk, readmission and phenotype prediction in patients with heart failure: exploring key data sources, input variables and outcomes
Source: BMC Med Inform Decis Mak. 2026 Jun 3;26:205. doi: 10.1186/s12911-026-03560-8 (PMC13235149; doi:10.1186/s12911-026-03560-8)
Supplement: Supplementary file 5 — Supplementary material 5 [file 12911_2026_3560_MOESM5_ESM.docx]

Additional file 1 - PRISMA 2020 checklist

**Data items**

In our review, we sought data on several key outcomes. We extracted the source, including author and publication year, as well as all algorithms considered in the studies. For each study, we identified the proposed algorithm or the one demonstrating the best performance metrics, specifically accuracy and AUC, which were used for comparison. Additionally, we collected information on the data source, the number of variables included in the AI analysis, and the most relevant variables highlighted as influential for predictions. This comprehensive approach ensured a standardized and thorough evaluation of all relevant outcomes.

**Synthesis methods and reporting bias**

For the extraction of data on accuracy and Area Under the Curve (AUC), only the values of the proposed algorithms were considered, thereby reflecting the best-performing results in each study. If predictions were made for multiple time frames, such as 30 days and 60 days, the respective values for accuracy and AUC were extracted for each period. In cases where performance metrics were missing, the notation "n.a." (not available) was applied.

To ensure clarity and consistency, assumptions were made only where the reporting of performance metrics was ambiguous, and these were documented transparently. This approach allowed for a comprehensive and standardized data extraction process while acknowledging the limitations posed by incomplete reporting in some studies.

To assess the risk of reporting biases, we reviewed the completeness of reported results in the included studies and cross-checked them for consistency. In cases of uncertainty, a second author independently evaluated the data to ensure objectivity and reduce the risk of bias.

**Reporting bias assessment**

We did not use formal tools to assess the risk of bias due to missing results. However, we carefully reviewed the included studies to identify any gaps or inconsistencies in reporting, such as missing performance metrics, descriptions of outcome variables, or the most relevant variables influencing predictions. Missing values for key metrics, such as accuracy, AUC, or relevant variables, were documented and marked as 'n.a.' (not available). This ensured transparency and allowed us to account for incomplete reporting during data synthesis and interpretation. Additionally, studies with comprehensive reporting were prioritized to minimize the potential impact of reporting bias.

**Study selection**

As part of the full-text analysis, publications were excluded if they were of insufficient length and thus lacked the required data or if they only compile statistics. In addition, publications were excluded if the outcome variable was not clearly defined or if the relevant parameters were not mentioned in the AI model used, as this would result in a lack of comparability of the results. Furthermore, publications were also excluded if the determination or diagnosis of heart failure was defined as the outcome variable or other target variables of the AI model. Although such studies focus on heart failure, the outcome variables of mortality, readmission and phenotyping are primarily considered for this analysis. In addition, articles that dealt with AI-related technologies such as blockchain or the Internet of Things, or that reviewed the application of monitoring systems or other systems using these technologies, were excluded. The following table shows some examples of the papers for the exclusion criteria:

| **Exclusion criteria** | **Author (year)** | **Title** |
| --- | --- | --- |
| Insufficient length | Hon et al. (2016) | Risk stratification for hospital readmission of heart failure patients: A machine learning approach |
| Outcome focused on heart failure diagnosis | Naghavi et al. (2024) | AI-enabled cardiac chambers volumetry in coronary artery calcium scans (AI-CACTM) predicts heart failure and outperforms NT-proBNP: The multi-ethnic study of Atherosclerosis |
| Focused on statistical analysis | Fassina et al. (2020) | A random shuffle method to expand a narrow dataset and overcome the associated challenges in a clinical study: A heart failure cohort example |
| Use of other technologies or systems | Umer et al. (2023) | Heart failure patients monitoring using IoT-based remote monitoring system |
| Use of other technologies or systems / ECG Data | Shrestha and Yu (2022) | ECG Data Analysis with IoT and Machine Learning |
